# Micronutrients and risks of three main urologic cancers: A mendelian randomization study

**DOI:** 10.3389/fnut.2023.1016243

**Published:** 2023-02-27

**Authors:** Yi Lu, Hao Su, Yutao Wang, Hongjun Li

**Affiliations:** Department of Urology, Peking Union Medical College, Peking Union Medical College Hospital, Chinese Academy of Medical Sciences, Beijing, China

**Keywords:** micronutrients, prostate cancer, renal cell carcinoma, bladder cancer, Mendelian randomization

## Abstract

**Background:**

The effect of micronutrients on urologic cancers has been explored in observational studies. We conducted the two-sample mendelian randomization (TSMR) study to investigate whether micronutrients could causally influence the risk of urologic cancers.

**Methods:**

Summary statistics for four micronutrients and three main urologic cancers outcomes were obtained from genome-wide association studies (GWAS). MR analyses were applied to explore the potential causal association between them. Sensitivity analyses using multiple methods were also conducted.

**Results:**

Genetically predicted one SD increase in serum copper and iron concentrations was causally associated with increased risks of renal cell carcinoma (RCC) (OR = 3.021, 95%CI = 2.204–4.687, *P* < 0.001, male; OR = 2.231, 95%CI = 1.524-3.953, *P* < 0.001, female; OR = 1.595, 95%CI = 1.310–1.758, *P* = 0.0238, male; OR = 1.484, 95%CI = 1.197–2.337, *P* = 0.0210, female, respectively) and per SD increase in serum zinc levels was related to decreased risks of RCC (OR = 0.131, 95%CI = 0.0159–0.208, *P* < 0.001, male; OR = 0.124, 95%CI = 0.0434–0.356, *P* < 0.001, female). No significant results were observed between micronutrients and the risk of bladder cancer after Bonferroni correction. Additionally, per SD increase in serum zinc level was associated with a 5.8% higher risk of prostate cancer (PCa) [OR = 1.058, 95%CI = 1.002–1.116, *P* = 0.0403, inverse-variance weight (IVW)].

**Conclusions:**

Micronutrients play a vital role in the development of urological tumors. Future studies are required to replicate the findings, explore the underlying mechanisms, and examine the preventive or therapeutic role of micronutrients in clinical settings.

## 1. Introduction

Urologic tumor refers to tumors that affect the organs and structures of the urinary system of both men and women and the reproductive system of men. Three most prevalent types of urologic tumors are: prostate cancer (PCa), renal cell carcinoma (RCC), and bladder cancer (BCa) (1). The incidence of kidney, bladder, and prostate cancers cases increased between 1990 and 2013 and mortality increased 1.6-fold during the same time period. Urologic cancer burden has increased globally amid population growth and aging (2). Efforts to expand the global oncologic workforce and reduce preventable factors may contribute to cancer management (3). Nowadays, several risk factors have been established, such as lipid composition, obesity, and cigarette, etc (4). However, the role of nutrition in urologic cancer development is still unclear.

Dietary trace metals, including zinc, copper, iron, and selenium, etc. have been shown to influence the risk of cancer through oxidative stress, DNA injury and repair, regulating cell cycle, and angiogenesis (5). Some observational studies using food frequency questionnaires (FFQs) indicated the anti-tumor role of nutrients or dietary intake of nutrients in urologic cancer, while these results are conflicting and concerns about potential biases from confounding factors can't be dispelled (6).

Mendelian randomization (MR) that uses genetic variants as instrumental variables is widely used in epidemiological studies to examine whether a potential factor could casually influence an outcome. Different from traditional observational studies, this method could dramatically lower the effect of confounders and reverse causation (7). Two-sample Mendelian randomization (TSMR), which belongs to MR methodology and uses two samples drawn from the same underlying population with no overlap of participants between the two samples, is a method to estimate the causal effect of an exposure on an outcome using only summary statistics from genome-wide association studies (GWAS). Some large-scale GWAS on micronutrients and urologic cancers have also been published, providing high-quality genetic instruments to conduct MR study (8, 9). These GWAS have been used and validated in several previous MR studies (10, 11). To fill in the gap, we conducted the TSMR study to identify the potential effect of microelement levels on urologic cancer risk.

## 2. Method

### 2.1. Study design

**Figure 1:**
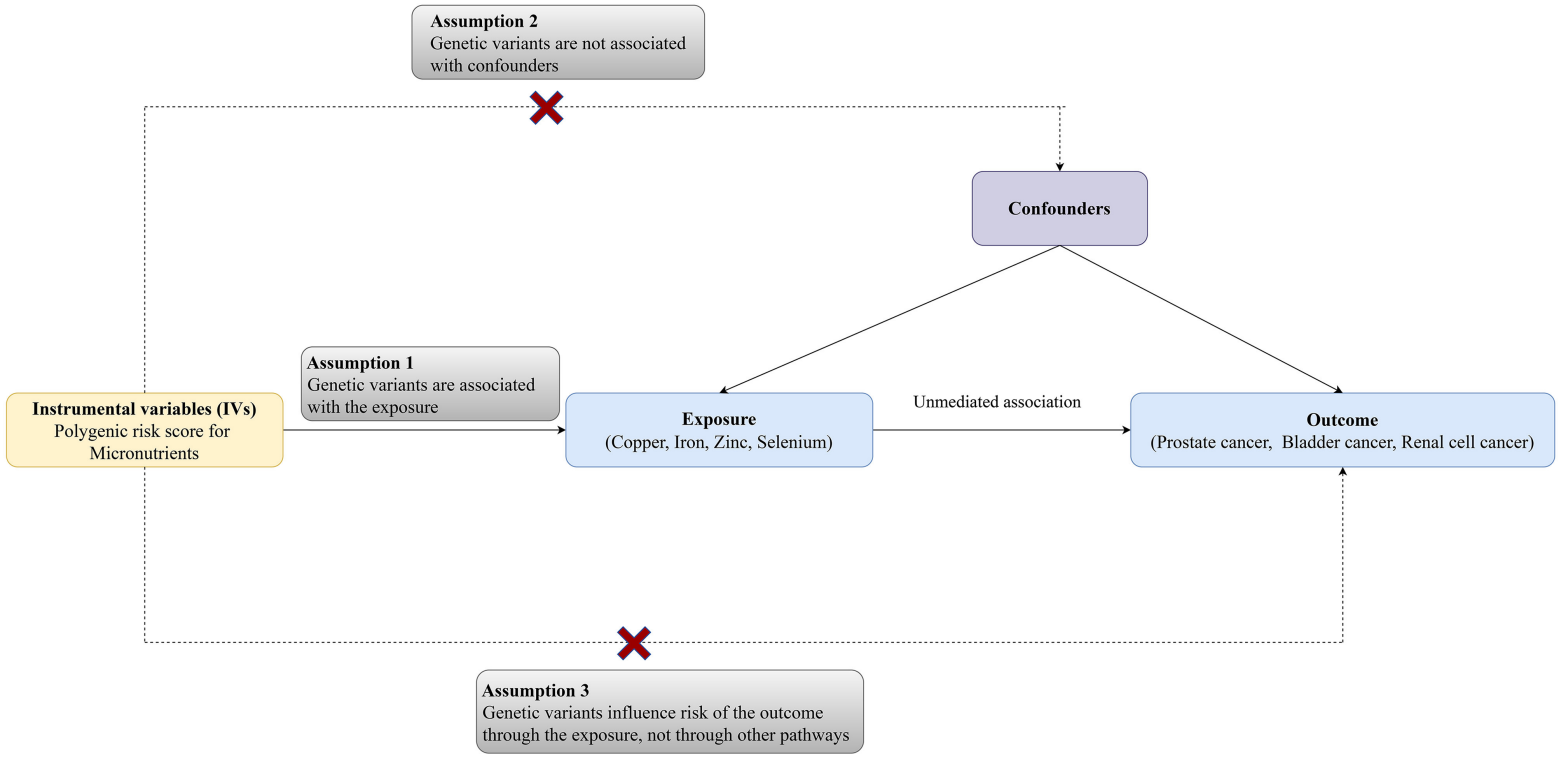
The diagram of the study.

The MR analysis was designed to evaluate the associations between microelement levels and risks of urologic tumors (RCC, BCa, and PCa). Single nucleotide polymorphisms (SNPs) for common microelements (Cu, Zn, Fe, and Se) were selected as instrumental variables from previously published genome-wide association study (GWAS) analyses. Three key assumptions need to be satisfied: (a) the SNPs should have strong associations with microelement levels; (b) the chosen SNPs should be independent of confounders; (3) the SNPs should affect cancer only *via* microelement levels. The diagram of the TSMR was shown in Figure 1 (12).

### 2.2. Data sources

The study utilized summarized genetic data from the Genetics of Iron Status (GIS) consortium (13), Prostate Cancer Association Group to Investigate Cancer Associated Alterations in the Genome (PRATICAL) (8), International Academic & Research Consortium (IARC) (14), and UK Biobank (UKB) (9). Details about the sources were shown in Table 1. The original GWAS had been approved by corresponding ethics committee, and the approval of current study was obtained from the Medical Research Ethics Committee of Peking Union Medical College Hospital.

**Table 1:** The characteristics of GWAS studies on the exposures and outcomes.

**Exposure**	**Consortium**	**Total population**	**Cases/controls**	**Ethnicity**	**References**
Copper	NA	2,603	NA	European	Genome-wide association study identifies loci affecting blood copper, selenium and zinc PubMed id: 23720494
Zinc	NA	2,603	NA	European	Genome-wide association study identifies loci affecting blood copper, selenium and zinc PubMed id: 23720494
Iron	GIS	23,986	NA	European	Novel loci affecting iron homeostasis and their effects in individuals at risk for hemochromatosis PubMed id: 25352340
Blood and toenail selenium	NA	4,162	NA	European	Genome-wide association Study of selenium concentrations. PubMed id: 25343990
Blood selenium	NA	2,603	NA	European	Genome-wide association study identifies loci affecting blood copper, selenium and zinc PubMed id: 23720494
**Outcome**	**Consortium**	**Total population**	**Cases/controls**	**Ethnicity**	**References**
Overall PCa	PRATICAL	140,254	79,148/61,106	European	Association analyses of more than 140,000 men identify 63 new prostate cancer susceptibility loci. PubMed id: 29892016
RCC in female	IARC	5,087	1,992/3,095	European	Sex-specific associations in genome-wide association analysis of renal cell carcinoma PubMed id: 31231134
RCC in male	IARC	8,143	3,227/4,916	European	Sex-specific associations in genome-wide association analysis of renal cell carcinoma PubMed id: 31231134
Bladder Cancer	UKB	462,933	1,101/ 461,832	European	UK Biobank: An Open Access Resource for Identifying the Causes of a Wide Range of Complex Diseases of Middle and Old Age PubMed id: 25826379

N.A., not available; GIS, Genetics of Iron Status; PRATICAL, Prostate Cancer Association Group to Investigate Cancer Associated Alterations in the Genome; IARC, International Academic and Research Consortium; UKB, UK Bioban.

### 2.3. Instrumental variable selection

Instrumental variable selection for Cu, Zn, and Se levels (serum), iron levels, and blood and toenail Se levels were based on a GWAS with 2,603 adults from Australia and the UK., a GWAS involving 48,972 individuals of European ancestry (GIS Consortium), and the UK Biobank study, respectively (13, 15, 16). Instrumental variables for RCC, BCa, and PCa were obtained from IARC (5,219 RCC cases and 8,011 controls of European ancestry), UKB (1,101 BCa cases and 461,832 controls of European ancestry), and PRATICAL consortium (79,148 PCa cases and 61,106 controls of European ancestry), respectively (8, 9, 14). Single nucleotide polymorphisms (SNPs) that met the locus-wide significance level (*P* < 10^−5^) and have genome-wide statistical significance (*P* < 5 × 10^−8^) were proposed as instrumental variables. Phenoscanner website was used to examine the pleiotropic effects of selected IVs and all used IVs were validated in previous studies (17, 18). All the SNPs selected in the study were shown in the Supplementary Table 1.

### 2.4. Study outcomes

RCC, BCa, and PCa were the outcomes. The latest GWAS involving the most complete available data on three types of cancers was selected. The sources were presented in Table 1.

### 2.5. Statistical analysis

Five different statistical methods were used to conduct the MR analyses. Firstly, the inverse-variance weight (IVW) approach was applied for the primary TSMR to quantify the causal associations between micronutrient (Cu, Fe, Se, and Zn) concentrations and the risk of three types of cancers (19). In the process, the ratio of coefficients was calculated to evaluate the causal effects. MR-Egger regression was used to examine the horizontal pleiotropy between IVs and three types of cancers, which adjusted micronutrients levels. Additionally, weighted median method (WM) only needs half of the effective SNPs was used as a supplement for the IVW approach (20). Finally, weighted mode and simple mode analyses were used to estimate the causal effect (21). https://shiny.cnsgenomics.com/mRnd/ was used for sample size test. This required four parts data: (a) Proportion of cases in the (intended) study; (b) Total sample size; (c) True odds ratio of the outcome variable per standard deviation of the exposure variable; (d) Proportion of variance in exposure variable explained by SNPs. Results indicated that with all the given sample size, analysis in each subgroup has strong statistical power (22). All traits related to screened SNPs were searched on the PhenoScanner website. Statistical analyses were performed repeatedly after removing confounder-related SNPs to improve the robustness and handle potential horizontal pleiotropy. Sensitivity analysis was also performed to assess whether some SNPs had a significantly independent influence on results *via* leave-one-out approach and the remaining estimate effect was shown when one SNP was excluded (23). The level of heterogeneity was estimated by using Cochran's Q statistics. All analyses were conducted in R software (version 4.1.2; http://www.rproject.org) with the “TwoSampleMR” package (version 0.5.6). Associations were considered as strong between micronutrients levels and cancer risks if they surpassed a stringent Bonferroni-corrected *P*-value threshold of 1.67 × 10^−3^ (0.05/3 cancer outcomes). The reporting of the MR study followed the existed rule (24).

## 3. Results

### 3.1. Associations between micronutrients and risk of RCC

For the four micronutrients, the primary estimate by IVW indicated that genetically predicted one SD increase in serum copper and iron concentrations was causally associated with increased risks of RCC (OR = 3.021, 95%CI = 2.204–4.687, *P* < 0.001, male; OR = 2.231, 95%CI = 1.524–3.953, *P* < 0.001, female; OR = 1.595, 95%CI = 1.310–1.758, *P* = 0.0238, male; OR = 1.484, 95%CI = 1.197–2.337, *P* = 0.0210, female, respectively) and per SD increase in serum zinc levels was related to decreased risks of RCC (OR = 0.131, 95%CI = 0.0159–0.208, *P* < 0.001, male; OR = 0.124, 95%CI = 0.0434–0.356, *P* < 0.001, female). However, no causal effect was observed in serum selenium and serum and toenail selenium (Table 2). Not all sensitivity analysis supported the causation between these micronutrients and RCC risk (Table 2 and Supplementary Figures 1–4).

**Table 2:** Two-sample MR estimates of relationship between genetically predicted micronutrients and cancer.

**Exposure**	**MR Method**	**Prostate cancer**			**Bladder cancer**			**Renal cell cancer (Female)**			**Renal cell cancer (Male)**		
		**No. of SNPs**	**OR (95% CI)**	***P*** **-Value**	**No. of SNPs**	**OR (95% CI)**	***P*** **-Value**	**No. of SNPs**	**OR (95% CI**	***P*** **-Value**	**No. of SNPs**	**OR (95% CI**	***P*** **-Value**
Copper	IVW	2	1.005 (0.952–1.061)	0.867	2	NA	NA	2	2.231 (1.524–3.953)	<0.001	2	3.021 (2.204–4.687)	<0.001
Iron	IVW	3	0.951 (0.896–1.011)	0.106	2	1.000 (0.999–1.001)	0.794	2	1.484 (1.197–2.337)	0.0210	2	1.595 (1.310–1.758)	0.0238
	MR-Egger	3	0.917 (0.814–1.034)	0.392	NA	NA	NA	NA	NA	NA	NA	NA	NA
	WM	3	0.954 (0.892–1.020)	0.170	NA	NA	NA	NA	NA	NA	NA	NA	NA
Zinc	IVW	2	1.058 (1.002–1.116)	0.0403	NA	NA	NA	2	0.124 (0.0434–0.356)	<0.001	2	0.131 (0.0159–0.208)	<0.001
	Wald ratio	NA	NA	NA	1	1.001 (1.000–1.002)	0.0841	NA	NA	NA	NA	NA	NA
Blood selenium	IVW	22	0.996 (0.975–1.017)	0.676	9	0.998 (0.997–0.999)	0.0317	22	0.790 (0.0779–8.030)	0.842	22	0.702 (0.0712–6.919)	0.762
	MR-Egger	22	1.023 (0.943–1.110)	0.595	9	0.999 (0.997–1.000)	0.560	22	1.070 (0.0991–1.9652)	0.166	22	3.858 (0.584–8.340)	0.203
	WM	22	1.003 (0.976–1.031)	0.835	9	1.000 (0.999–1.000)	0.0612	22	1.851 (0.918–2.977)	0.201	22	2.135 (1.940–2.387)	0.073
Blood and toenail selenium	IVW	12	0.985 (0.946–1.025)	0.458	4	0.999 (0.998–1.002)	0.182	12	0.175 (0.004–8.244)	0.375	11	0.0960 (0.00173–5.319)	0.253
	MR-Egger	12	1.044 (0.912–1.196)	0.545	4	1.000 (0.997–1.003)	0.213	12	0.08715 (0.0052–0.1597)	0.0257	11	1.7553 (0.4133–2.9804)	0.0570
	WM	12	0.985 (0.946–1.025)	0.452	4	0.999 (0.998–1.000)	0.986	12	0.224 (0.085–0.397)	<0.001	11	0.283 (0.0173–0.537)	0.0408

OR, odds ratio; CI, confidential interval; IVW, inverse-variance weight; WM, weighted median; SNP, single nucleotide polymorphisms; N.A., not available.

### 3.2. Associations between micronutrients and risk of BCa

**Figure 2:**
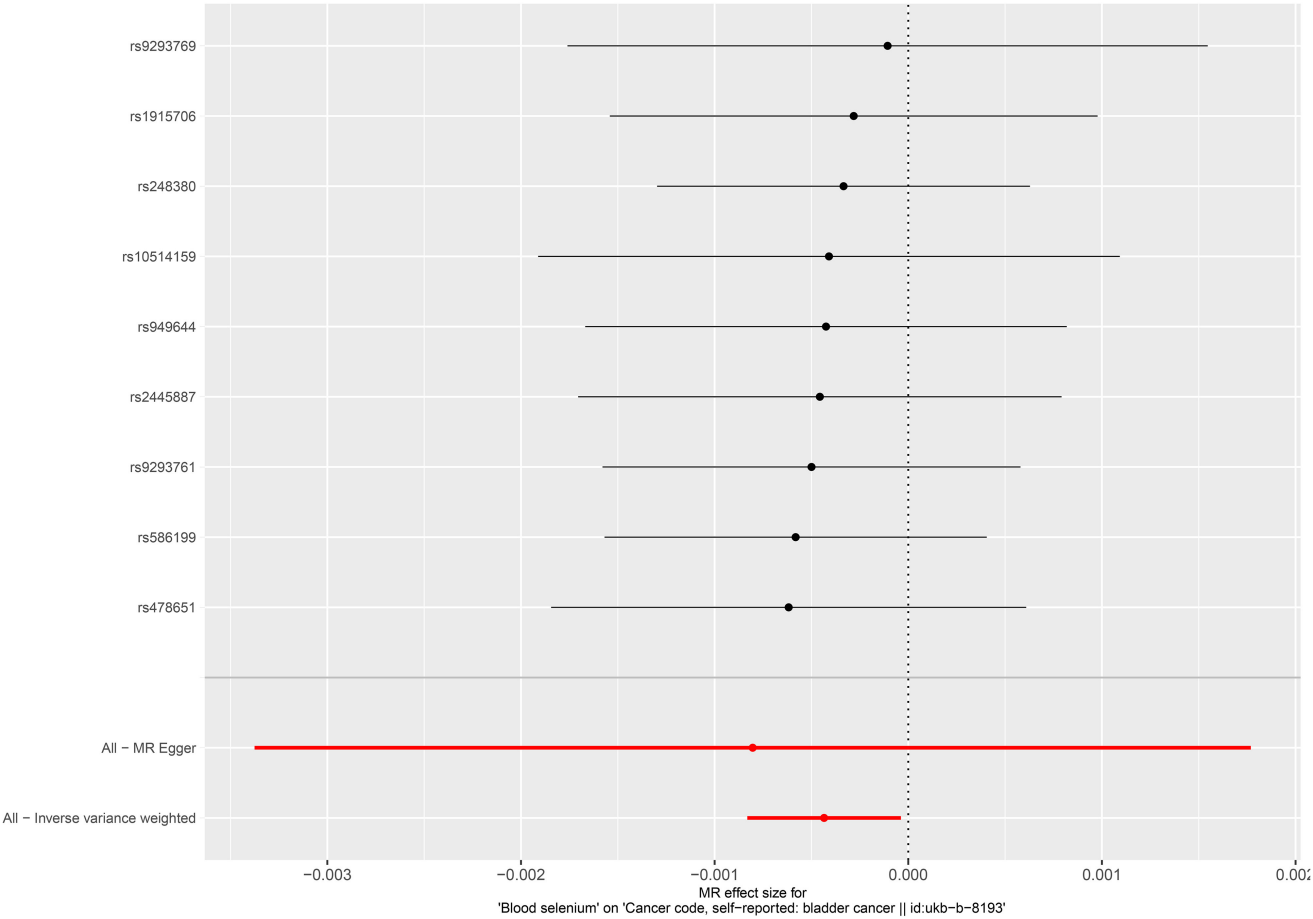
Forest plot for the association between circulating selenium and the risk of bladder cancer.

**Figure 3:**
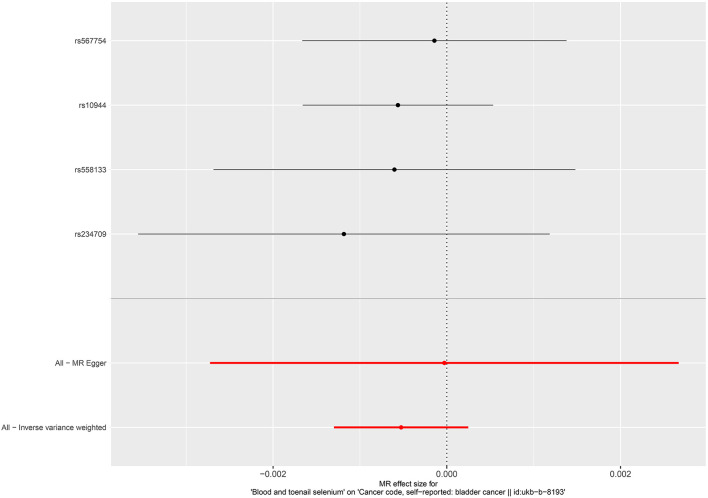
Forest plot for the association between blood and toenail selenium and the risk of bladder cancer.

No causal associations were observed between risks of BCa and serum iron level (OR = 1.000, 95%CI = 0.999–1.001, *P* = 0.794, IVW), zinc level (OR = 1.001, 95%CI = 1.000–1.002, *P* = 0.0841, Wald ratio), serum selenium (OR = 0.998, 95%CI = 0.997–0.999, *P* = 0.0317, IVW), and blood and toenail selenium (OR = 0.999, 95%CI = 0.998–1.002, *P* = 0.182, IVW) (Figures 2, 3). Sensitivity analyses revealed consistent results (Table 2 and Supplementary Figures 5–8).

### 3.3. Associations between micronutrients and risk of PCa

**Figure 4:**
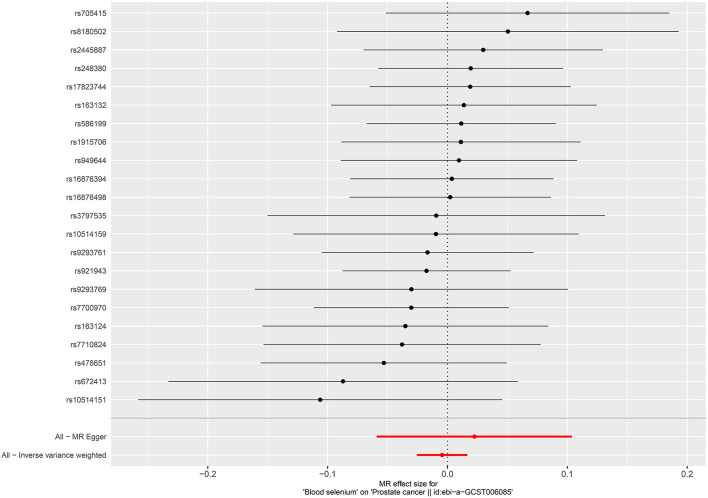
Forest plot for the association between circulating selenium and the risk of prostate cancer.

**Figure 5:**
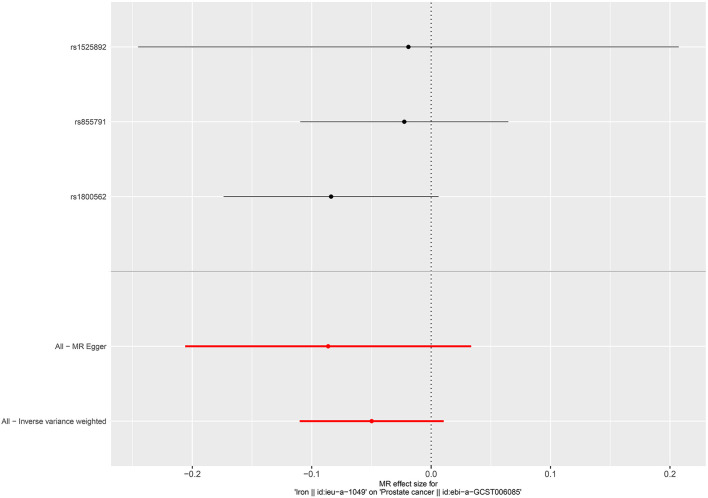
Forest plot for the association between circulating iron and the risk of prostate cancer.

**Figure 6:**
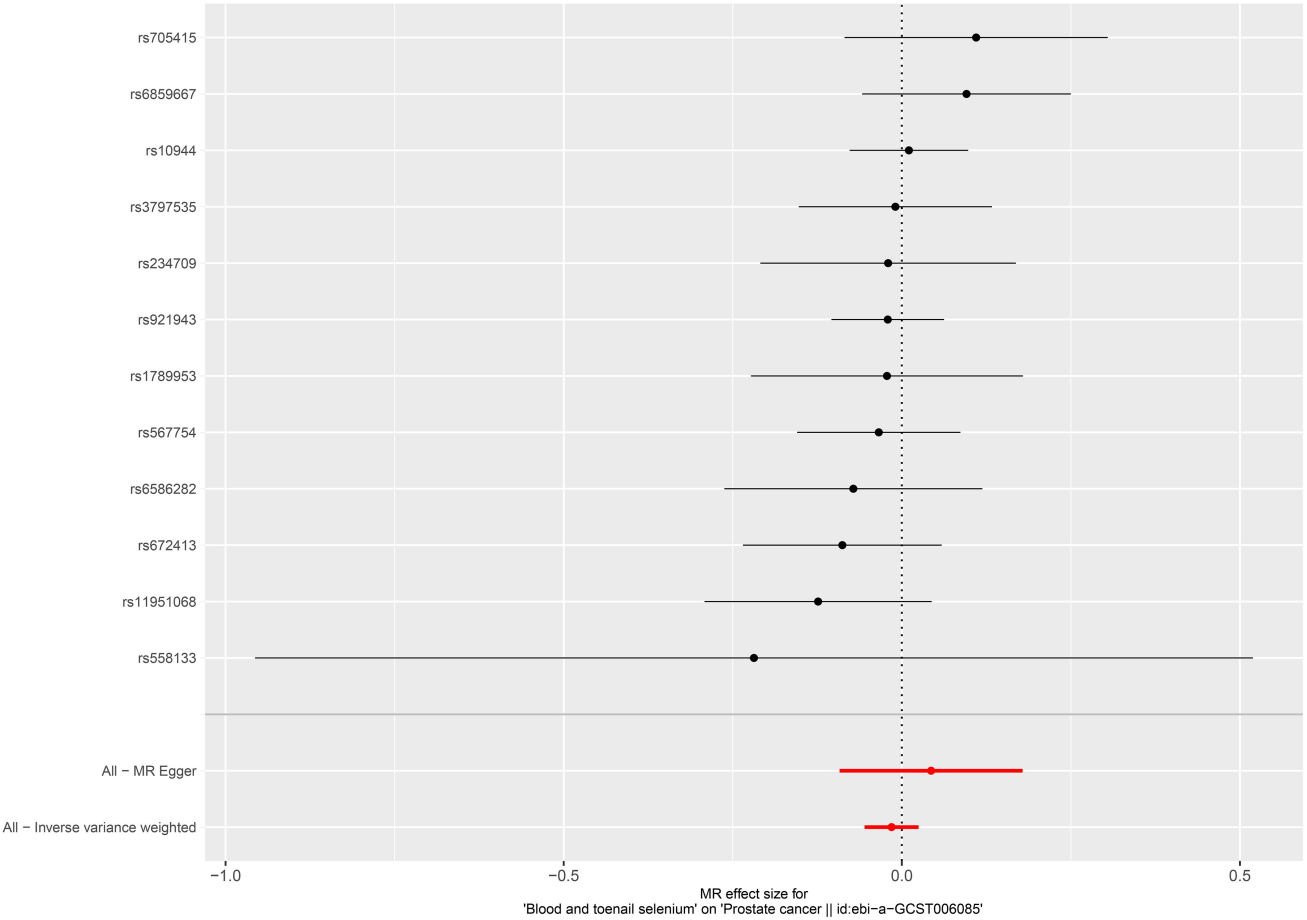
Forest plot for the association between blood and toenail selenium and the risk of prostate cancer.

Per SD increase in serum zinc level was associated with a 5.8% higher risk of PCa (OR = 1.058, 95%CI = 1.002–1.116, *P* = 0.0403, IVW). No causal associations were observed between risks of PCa and serum copper level (OR = 1.005, 95%CI = 0.952–1.061, *P* = 0.867, IVW), iron level (OR = 0.951, 95%CI = 0.896–1.011, *P* = 0.106, IVW), serum selenium (OR = 0.996, 95%CI = 0.975–1.017, *P* = 0.676), and blood and toenail selenium (OR = 0.985, 95%CI = 0.946–1.025, *P* = 0.458, IVW) (Figures 4–6). Consistent results were also achieved in sensitivity analysis (Table 2 and Supplementary Figures 9–14).

## 4. Discussion

In the current study, we investigated the causal effects of four micronutrients (Copper, Iron, Selenium, and Zinc) on risks of 3 urological tumors. The findings indicated that genetically increased Zinc levels were related to increased risk of PCa, while reduced risk of RCC. Furthermore, increased Copper and iron level were associated with increased risk of RCC. In terms of BCa, no causal effects were observed.

Prostate cancer is a common malignancy that affects millions of men globally (1). Prior observational and mendelian studies have indicated that serum calcium and selenium levels were not associated with the risk of PCa (10, 11), which was consistent with our results. A former study indicated that decreased zinc or increased copper level might play important role in the initiation of PCa, while no mendelian studies ever investigated the genetic causation between them (25). We found that increased serum zinc level was causatively related to increased risk of PCa, which was in accordance with a population-based study (26). A possible explanation for the phenomenon is the redistribution of zinc, leading to increased serum zinc and reduced intracellular zinc in prostate (27). However, no GWAS about intracellular zinc were available, the underlying mechanisms require further investigation.

RCC is another common urologic malignancy (1). No mendelian study ever investigated the effect of micronutrients on the risk of RCC. Previous studies focus on exploring the role of zinc-finger protein in RCC (28, 29), demonstrating that these zinc-finger proteins could suppress the proliferation, invasion and improve long-term prognosis. Only one study indicated that Zn in the medulla was significantly lower in RCC cases than in controls (30). The result is consistent with what we found. Greene et al. demonstrated that RCC development is commonly represented by accumulated iron and Wu et al. conducted an *in vivo* study that indicated that STEAP3 played a crucial role in the iron dysfunction in ccRCC (31, 32). Few clinical studies had showed the association between iron level and ccRCC risk in humans to date. Sridhar et al. indicated that a significantly higher copper concentration is noted in the blood and urine in RCC patients as compared to healthy controls (33). In accordance with this, we found that increased serum copper levels could genetically increase the risk of RCC. It might be attributed to oxidative stress responses to accumulation of heavy metals, while the exact underlying mechanism requires further studies.

For bladder cancer, former studies indicated that increased copper or zinc levels in the blood of patients were associated with angiogenesis in BCa and the risk of BCa (34, 35). However, we found no genetic associations between copper or zinc levels and BCa risk. Two reasons might explain the difference. Firstly, the small number of available SNPs may cause statistical biases. Secondly, the results of former observational studies were influenced by confounders. More GWAS and experimental studies are warranted.

Two uncommon diseases featured by the pathological accumulation of micronutrients should be mentioned. Wilson disease (WD) is an inherited disorder of copper metabolism, which is caused by homozygous or compound heterozygous mutations (the presence of two different mutant alleles) in *ATP7B* (36). Copper absorbed from the diet and copper released from hepatocytes with exhausted endogenous copper storage capacity progressively accumulate in other organs, most notably in the brain, eyes, kidneys, bones, and heart, exerting extrahepatic toxicity. Almost 90% of patients with WD has reduced level of serum copper and copper is mainly accumulated in organs. The most frequently reported cancer in WD patients is hepatocellular carcinoma (37). Few studies have reported WD patients complicated with urologic cancers, while the anti-copper therapy has been used and verified as a validated treatment in several cancers (38, 39). Haemochromatosis is a systemic iron overload of genetic origin, caused by a reduction in the concentration of the iron regulatory hormone hepcidin, or a reduction in hepcidin-ferroportin binding. Similar to WD, haemochromatosis featured by the accumulation of iron in the liver, is associated with elevated serum ferritin and increased serum transferrin saturation rather than serum iron level and it is mostly reported to have an association with hepatocellular carcinoma (40). The association between the two diseases and urologic tumors still requires further investigation.

Our findings have some clinical and research implications. Firstly, we firstly indicated the genetic associations between micronutrients and three main urologic tumors by using mendelian randomization. Some of the micronutrients we identified in this study can be used as cancer biomarkers for risk prediction. While the prerequisite to achieving this is the clear association between the serum micronutrient level and cancer risks (linear or U-shape or …). Based on this, we can further make a classification strategy, for example, using the median level as the cut-off. Given current evidence, there is still a long way to go. Secondly, appropriate therapy that could adjust micronutrient levels in the blood will contribute to the prevention of urologic tumors and, eventually, of the cancer-associated disease burden and mortality. However, it should be noted that no clear evidence (the number of RCTs is <20) about the micronutrient intervention and cancer risk or cancer progression can be found. While more research is needed to assess whether micronutrients may modify the risk of cancer in individuals with a specific genetic background or nutritional status, and to investigate possible differential effects of various forms of micronutrients. Thirdly, the bias caused by limited numbers of SNPs should be validated in experimental studies. Fourthly, the conflicting findings on the effect of Zinc on PCa and RCC should be examined in experimental studies. According to currently available literature, we supposed that different zinc-related protein expressions in the kidney and the prostate might play a role in the development of the two cancers. Moreover, the balance between the zinc influx protein family and zinc efflux transporters on different organs might make a difference (27).

Our study has some strengths. Firstly, the study was the first MR study to investigate the casual association between micronutrient levels and the risk of urologic tumors. Effects of confounders in observational studies are avoided. Secondly, all the included individuals were of European-descent, which could minimize the potential bias from population stratification. Additionally, four common micronutrients and three main urologic tumors were analyzed, which is comprehensive and informative. Finally, limitations should be pointed out. Firstly, findings achieved from the MR study consisted of European-descent population limited the generativity to other races. Secondly, serum micronutrients might have associations with nutrition status, intelligence, income, and education level, etc (41). All these factors might play as a confounder between micronutrients and urologic tumors, while a concrete role of these factors was not the aim of the study and it requires further research.

In conclusion, we found that genetically increased Zinc levels were related to increased risk of PCa, while reduced risk of RCC. Furthermore, increased Copper and iron level were associated with increased risk of RCC, no causal effects were observed in BCa. The results indicate that micronutrients play a vital role in urological tumors. Future studies are therefore warranted to validate our findings and examine whether micronutrient concentration surveillance or supplements could be potential interventions for urologic cancer prevention and treatment.

## Data availability statement

The original contributions presented in the study are included in the article/Supplementary material, further inquiries can be directed to the corresponding author.

## Ethics statement

Written informed consent was obtained from the individual(s) for the publication of any potentially identifiable images or data included in this article.

## Author contributions

YL, YW, and HL: conception and design. HL: administrative support. YL, HS, and YW: collection and assembly of data. YW and HS: data analysis and interpretation. All author manuscript writing and final approval of manuscript.
