# Supplementary material for: Micronutrients and risks of three main urologic cancers: A mendelian randomization study
Source: Front Nutr. 2023 Feb 27;10:1016243. doi: 10.3389/fnut.2023.1016243 (PMC10009189; doi:10.3389/fnut.2023.1016243)
Supplement: Supplementary file 1 [file Data_Sheet_1.PDF]

Supplementary Material

Supplementary Figures

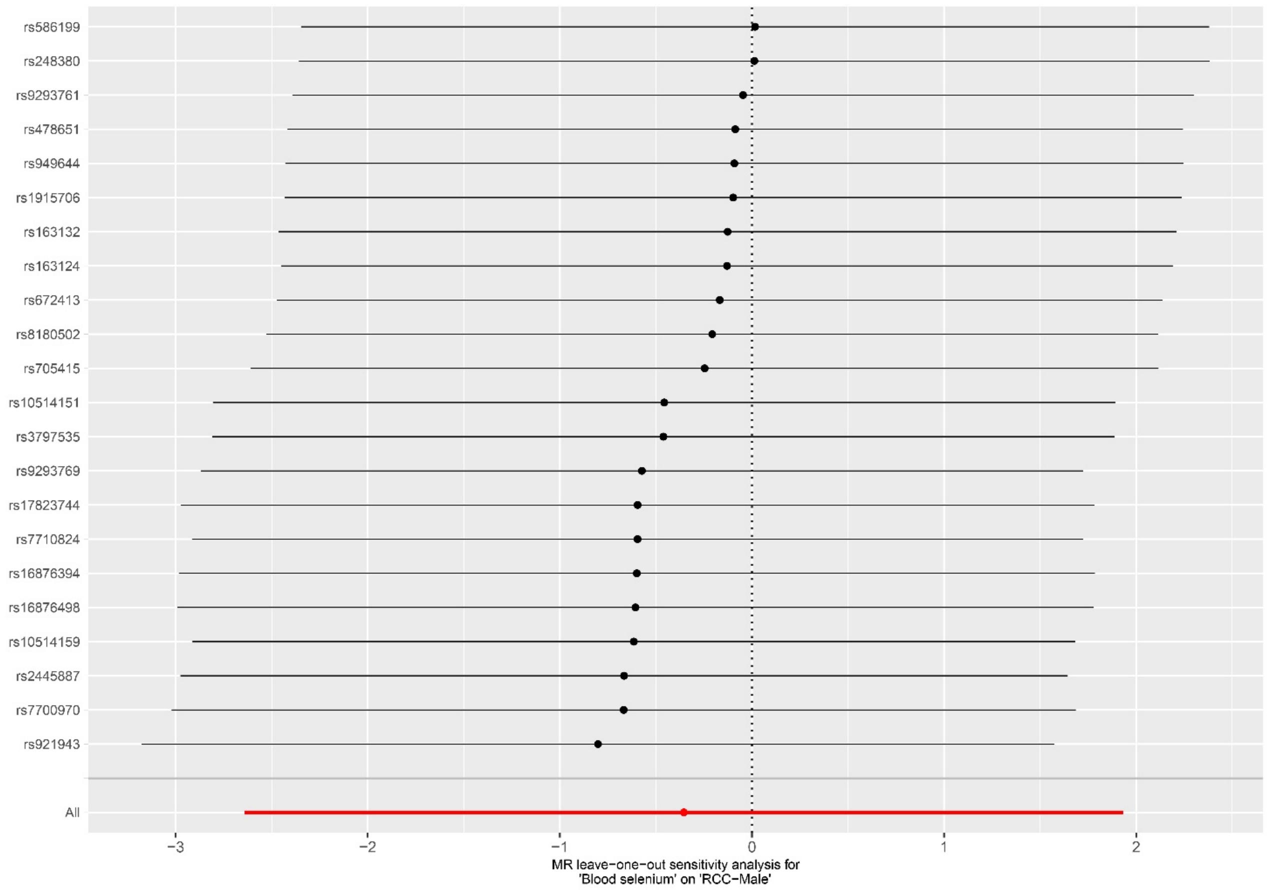

Supplementary Figure 1. Sensitivity analysis for the association between circulating selenium and the risk of renal cell carcinoma (male).

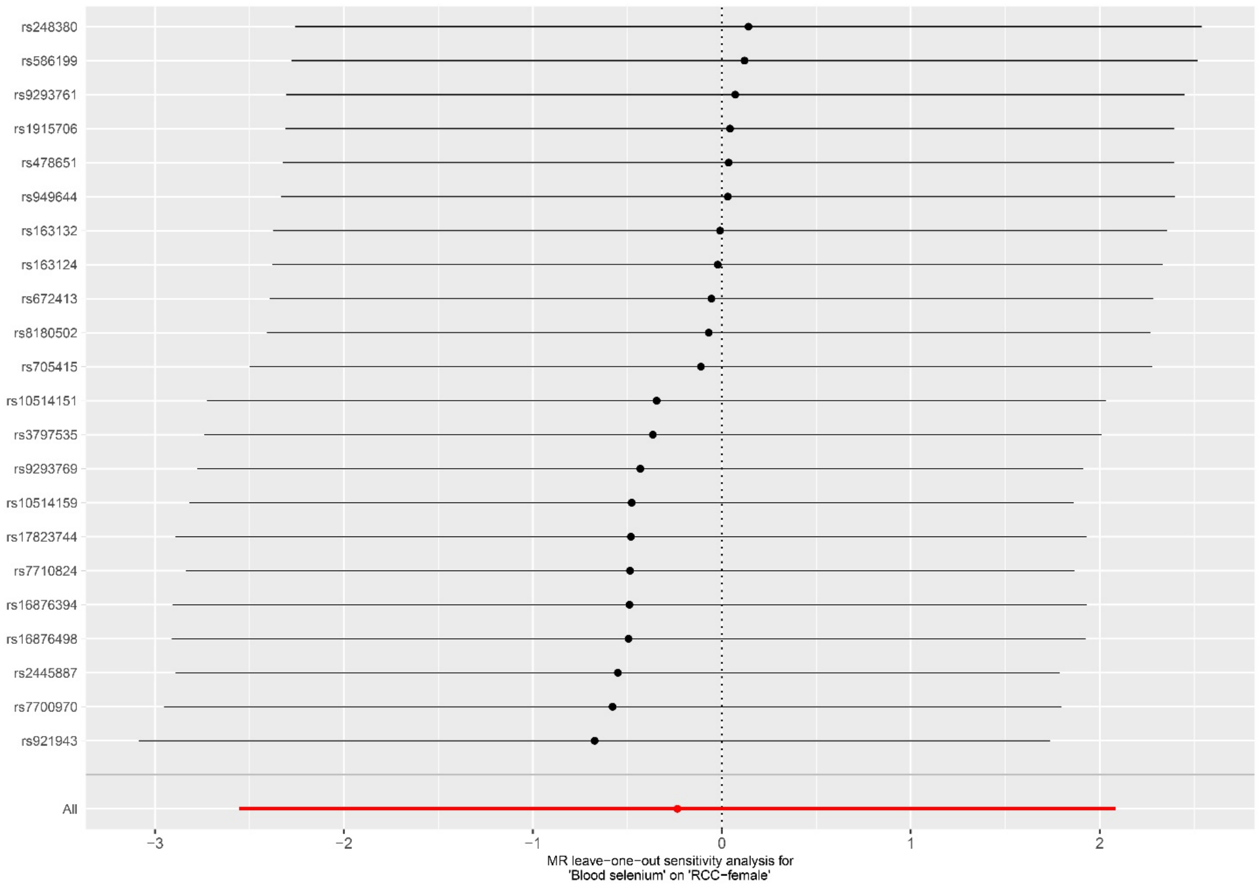

Supplementary Figure 2. Sensitivity analysis for the association between circulating selenium and the risk of renal cell carcinoma (female).

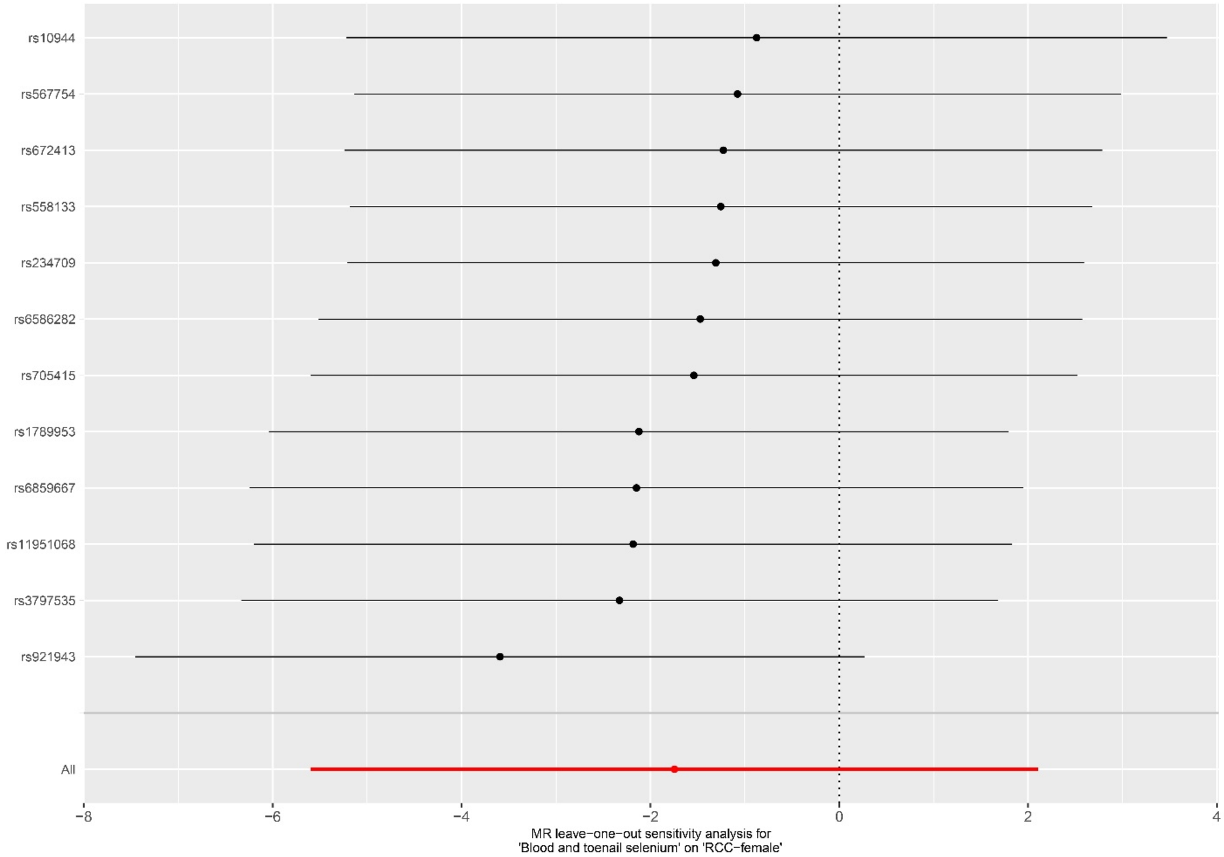

Supplementary Figure 3. Sensitivity analysis for the association between blood and toenail selenium and the risk of renal cell carcinoma (female).

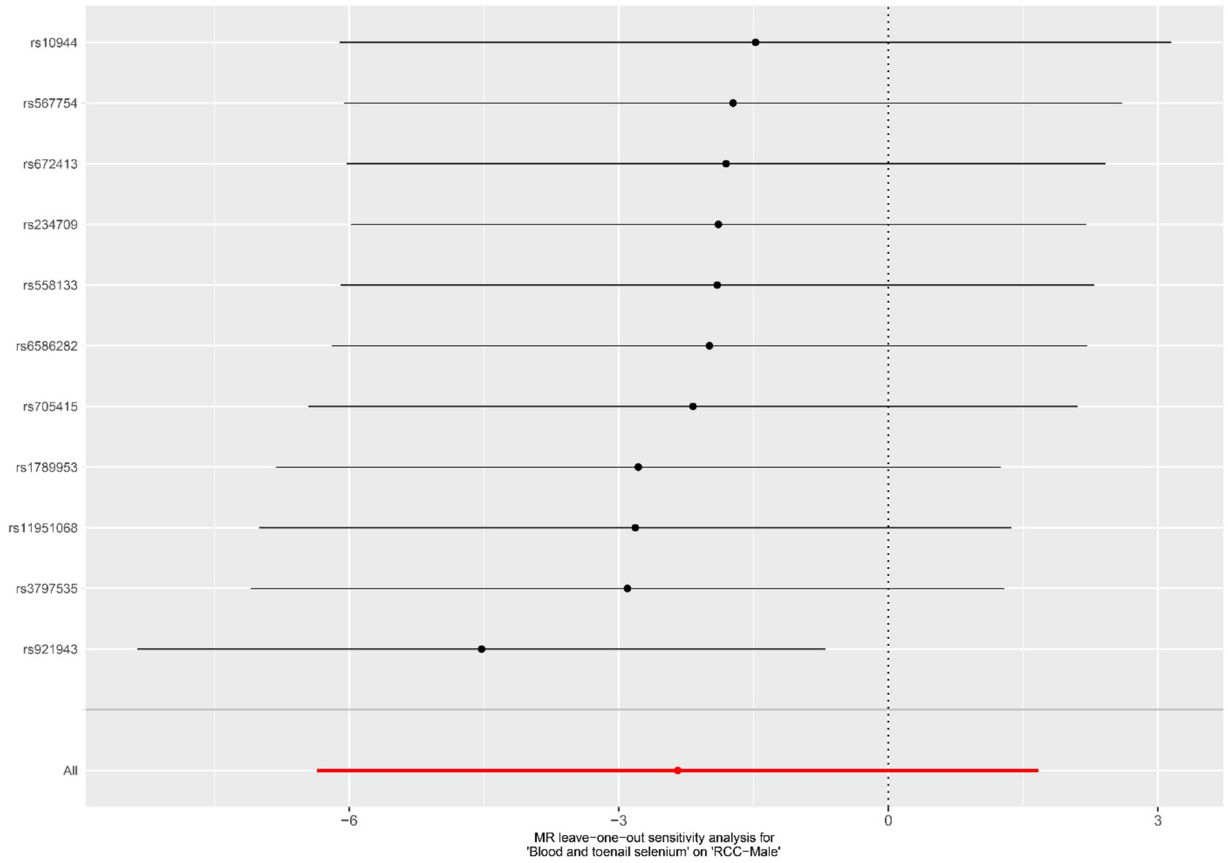

Supplementary Figure 4. Sensitivity analysis for the association between blood and toenail selenium and the risk of renal cell carcinoma (male).

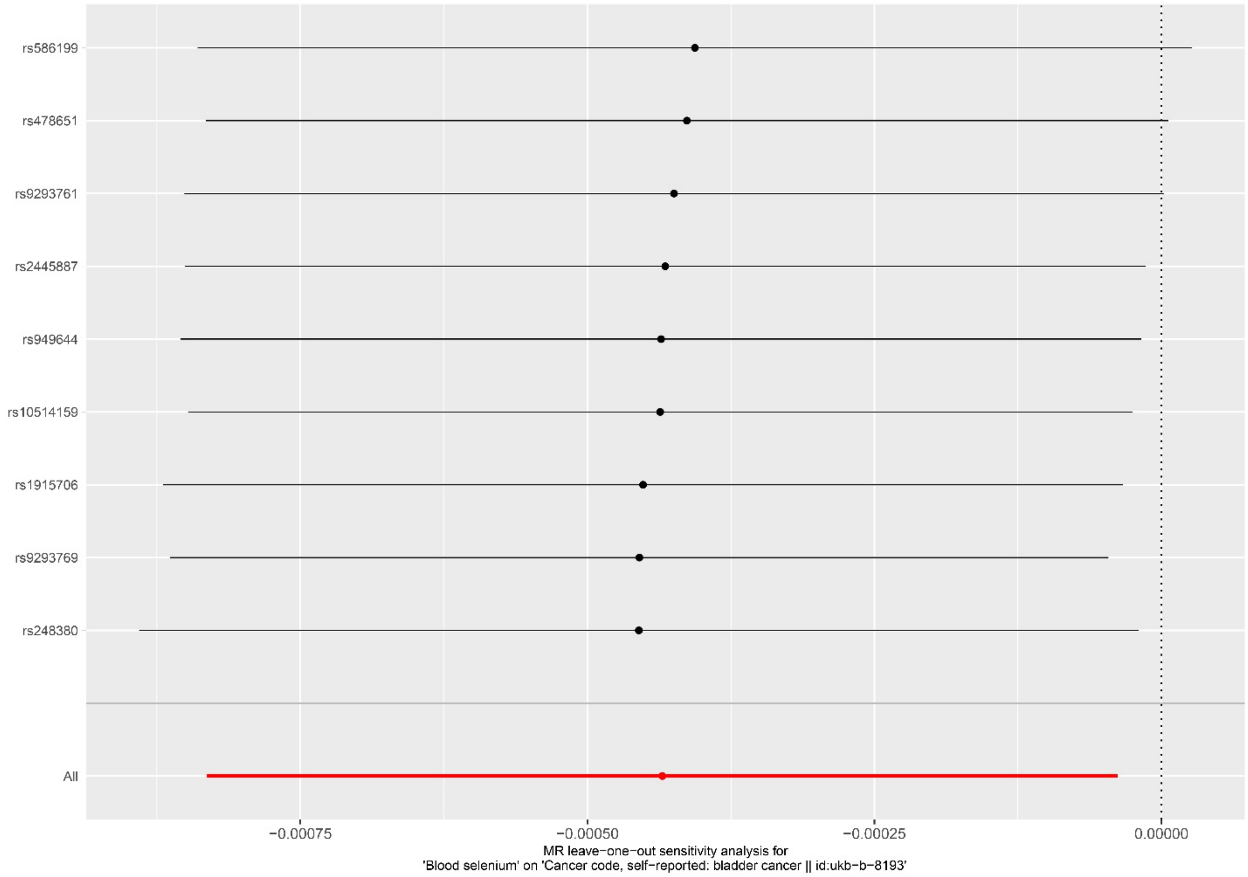

Supplementary Figure 5. Sensitivity analysis for the association between circulating selenium and the risk of bladder cancer.

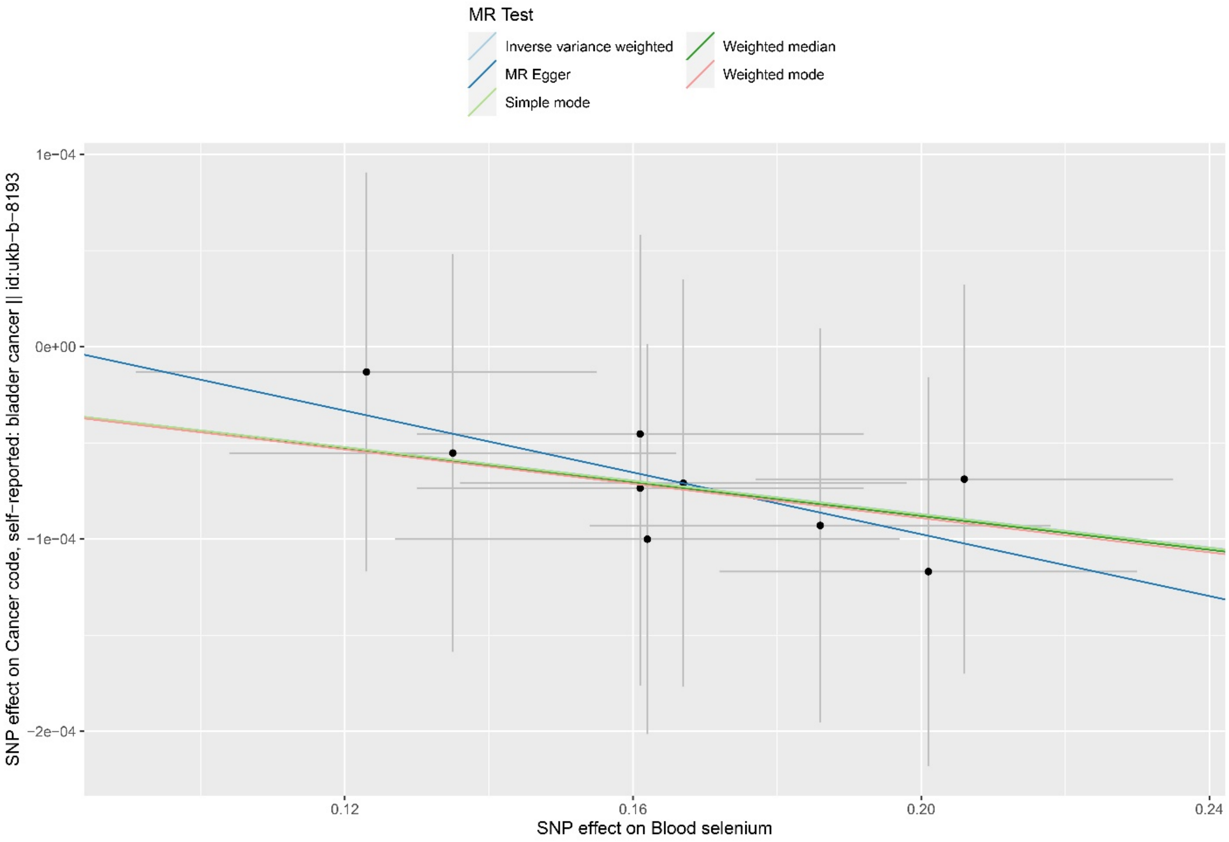

Supplementary Figure 6. Scatter plot of separate effects of SNPs for circulating selenium on bladder cancer risk.

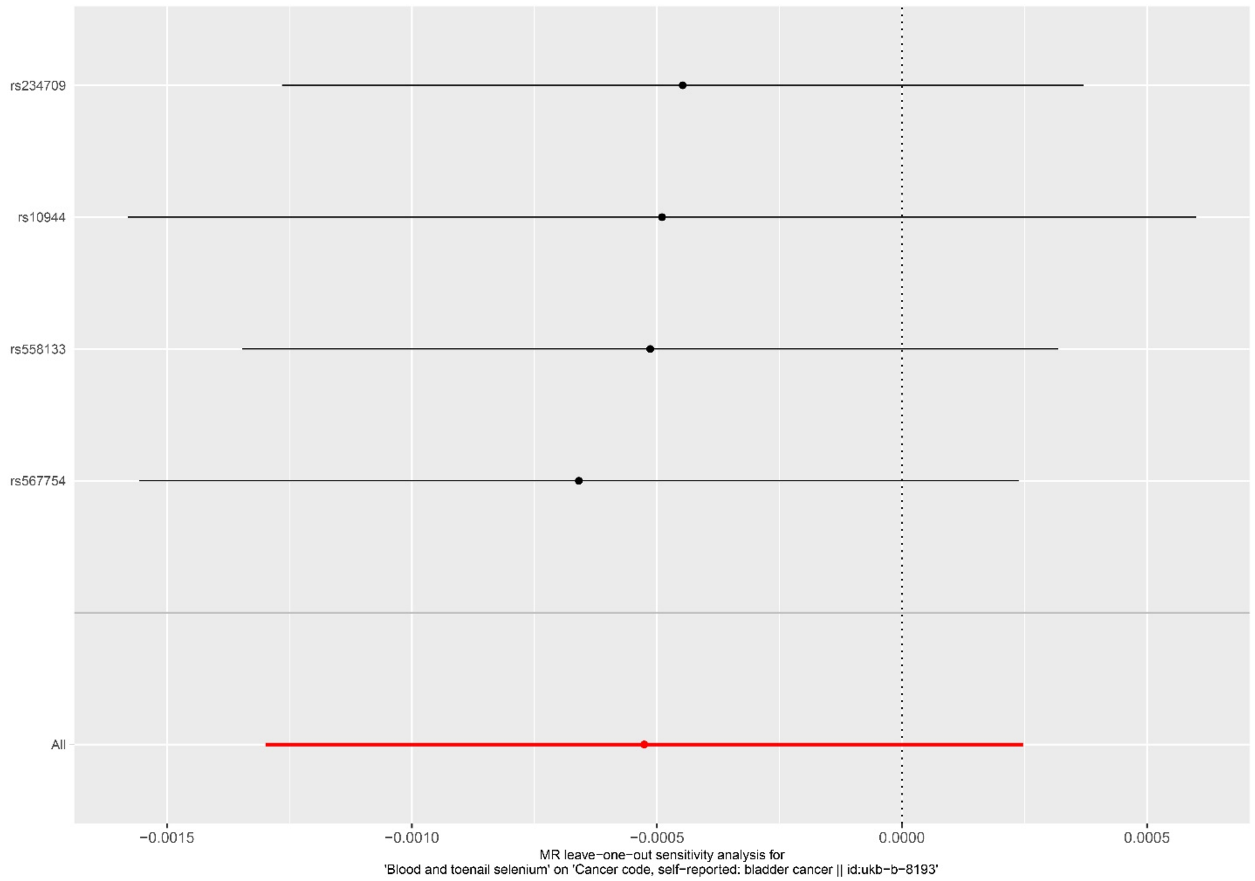

Supplementary Figure 7. Sensitivity analysis for the association between blood and toenail selenium and the risk of bladder cancer.

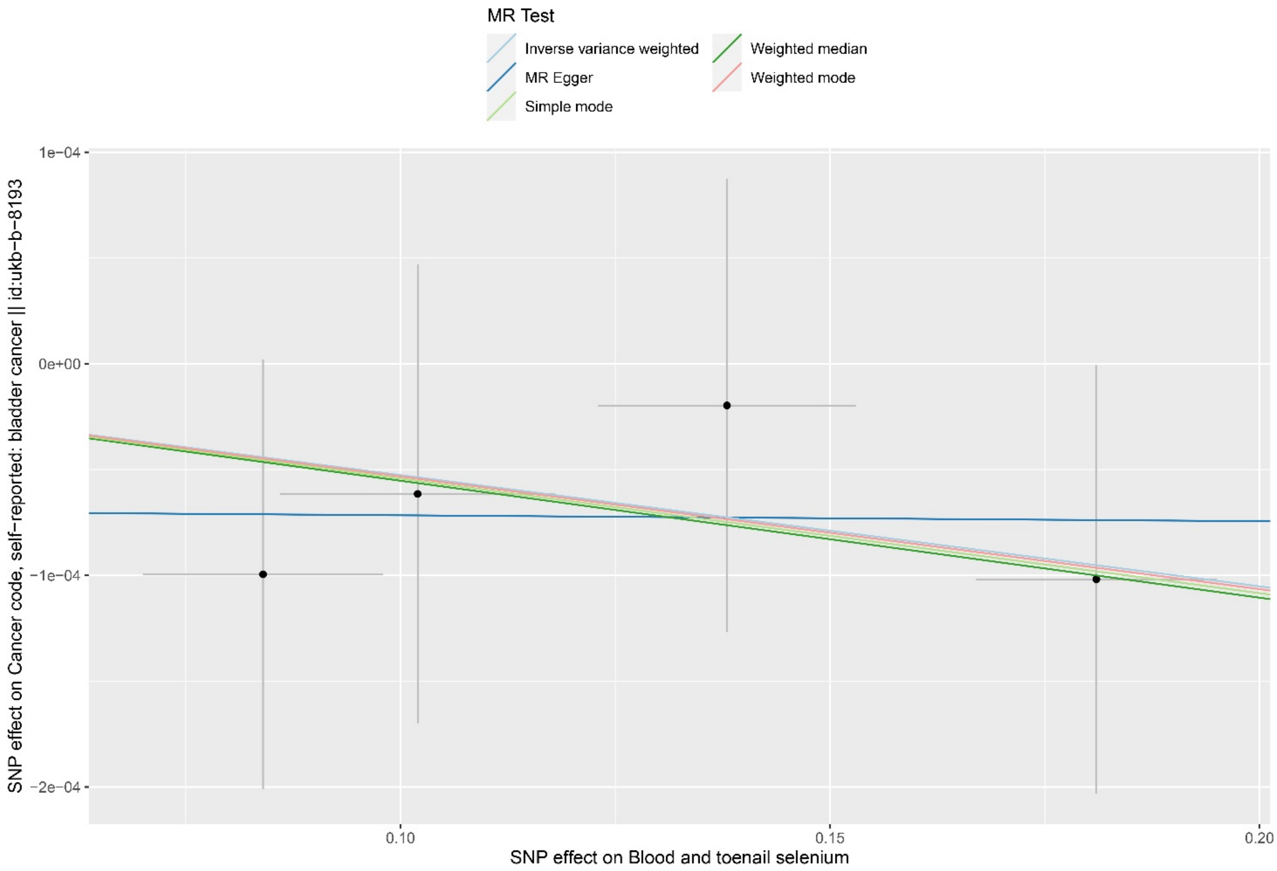

Supplementary Figure 8. Scatter plot of separate effects of SNPs for blood and toenail selenium on bladder cancer risk.

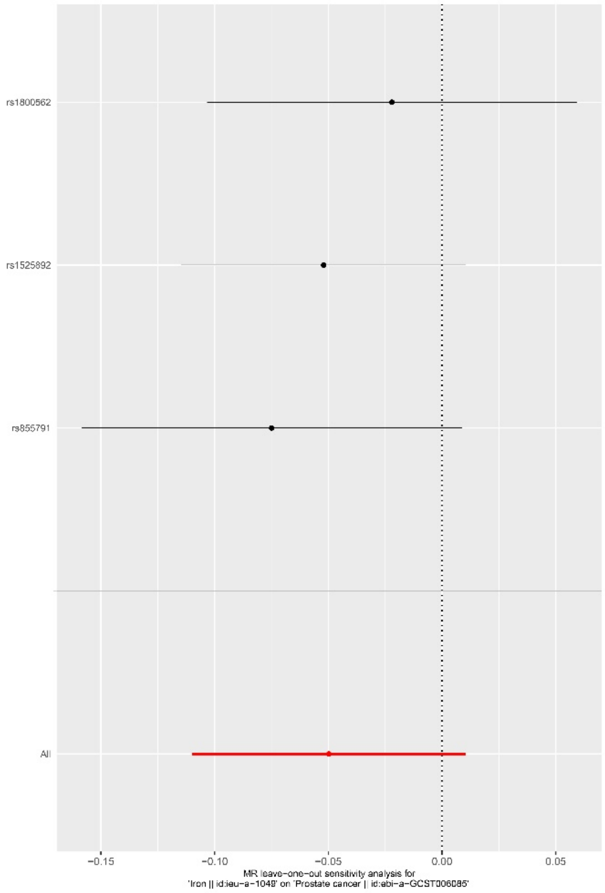

Supplementary Figure 9. Sensitivity analysis for the association between circulating iron and the risk of prostate cancer.

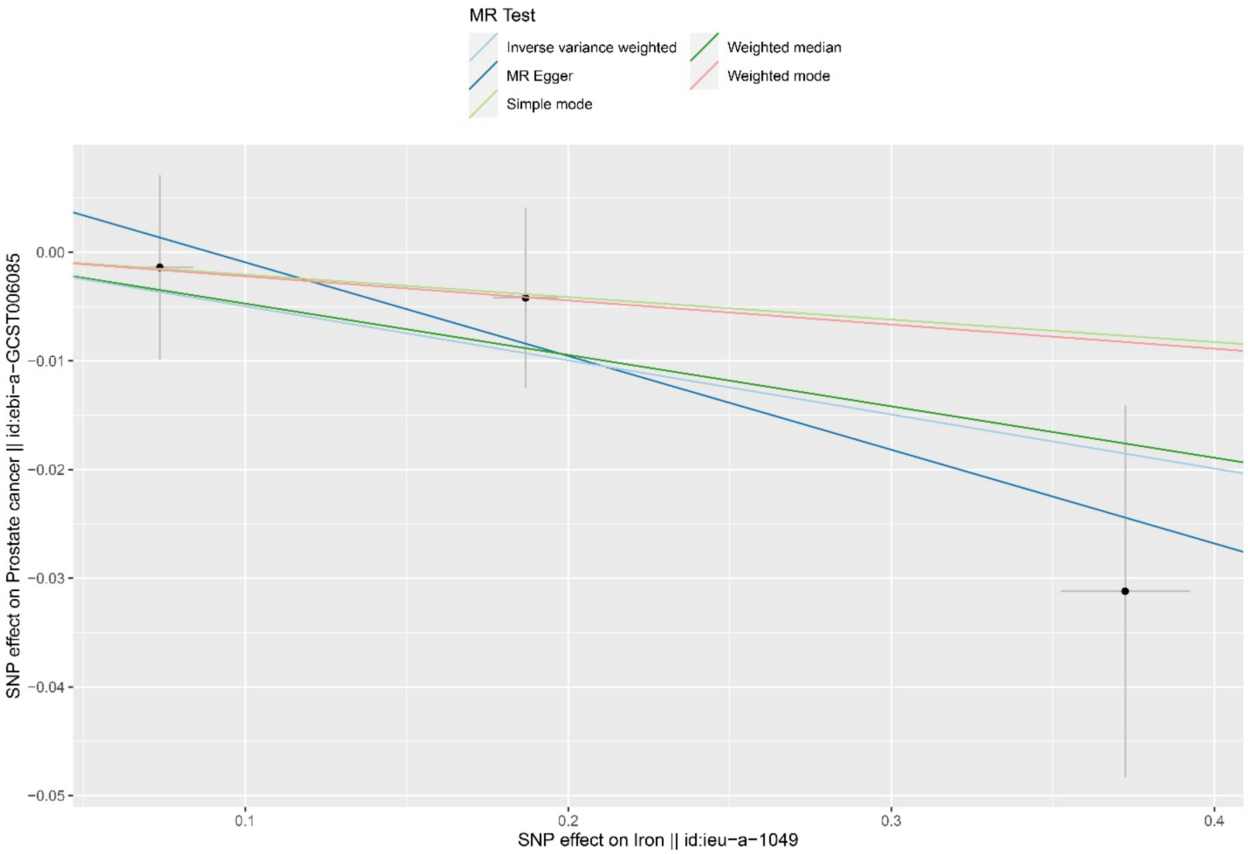

Supplementary Figure 10. Scatter plot of separate effects of SNPs for circulating iron on prostate cancer risk.

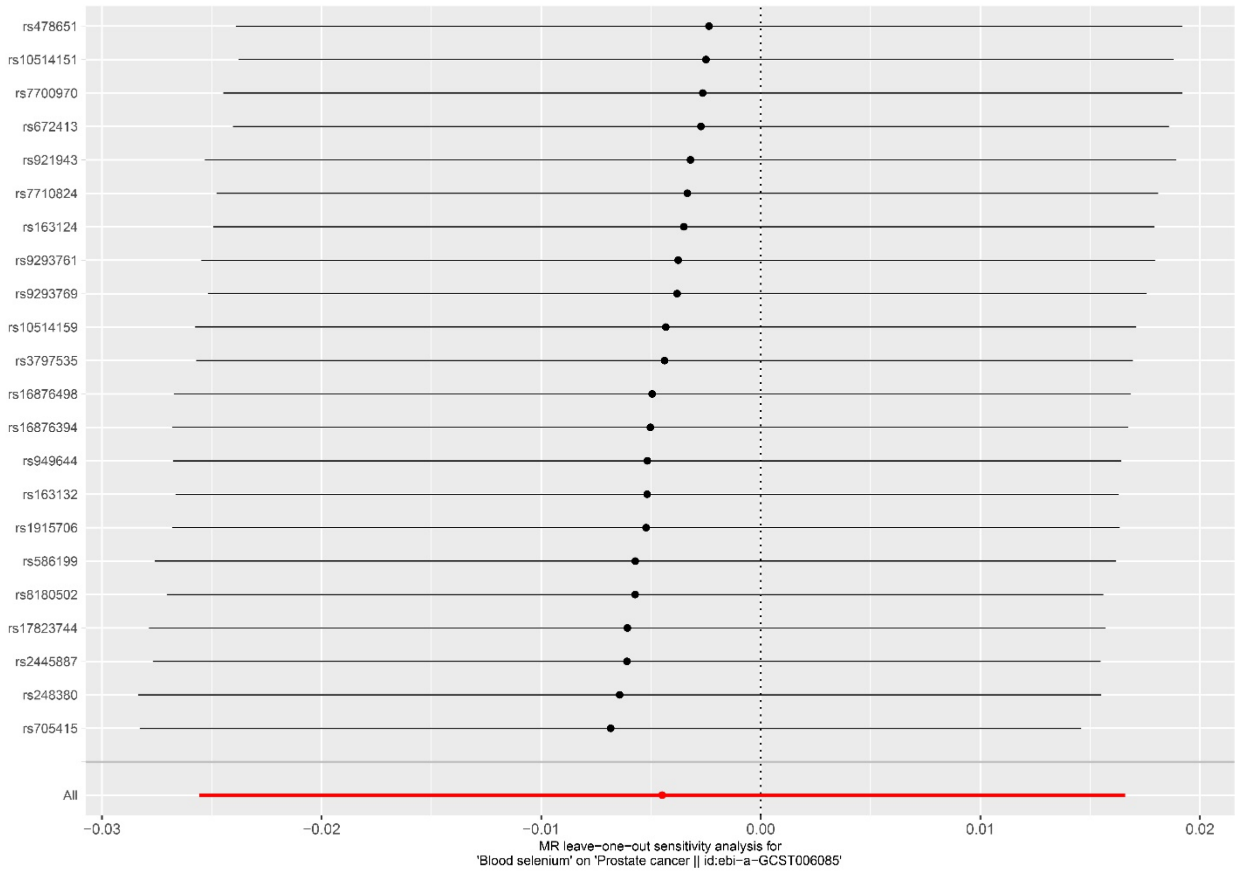

Supplementary Figure 11. Sensitivity analysis for the association between circulating selenium and the risk of prostate cancer.

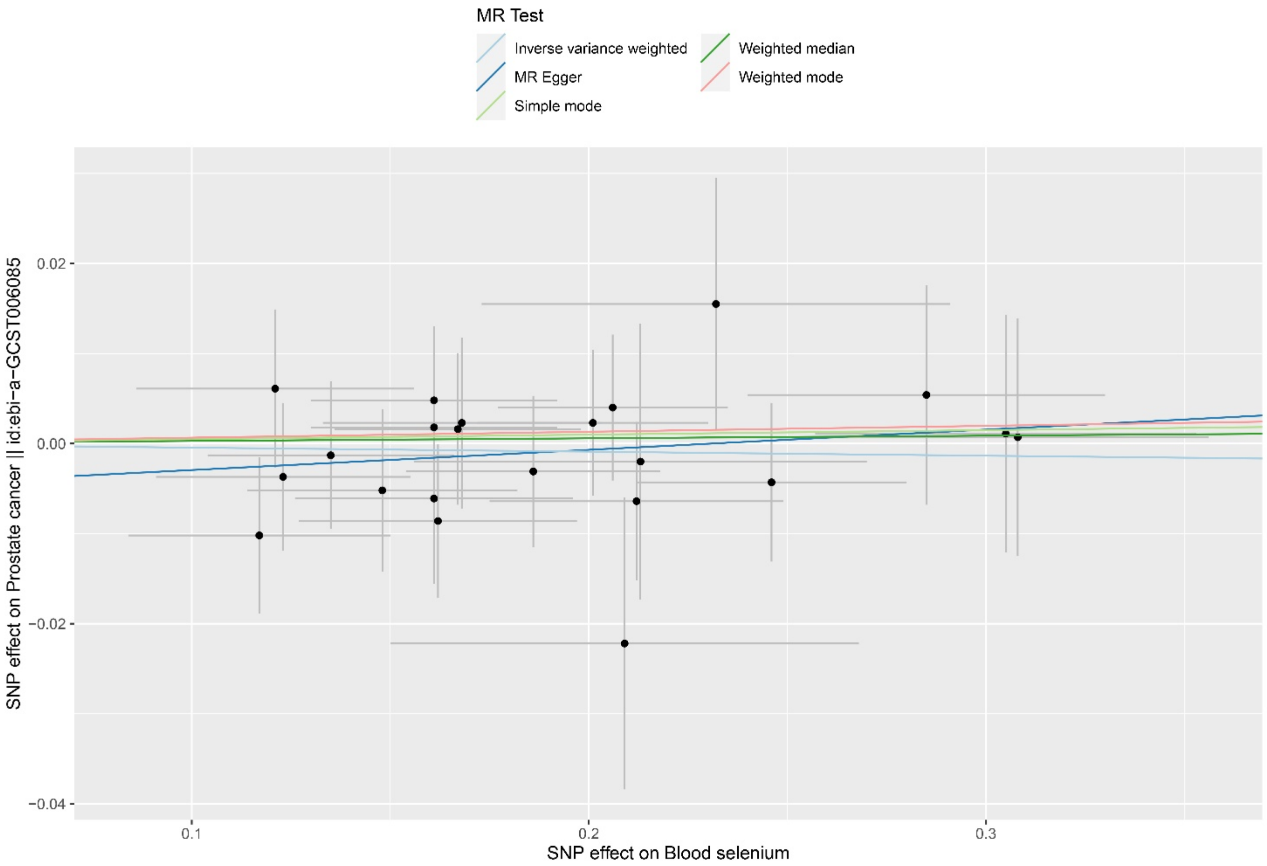

Supplementary Figure 12. Scatter plot of separate effects of SNPs for circulating selenium on prostate cancer risk.

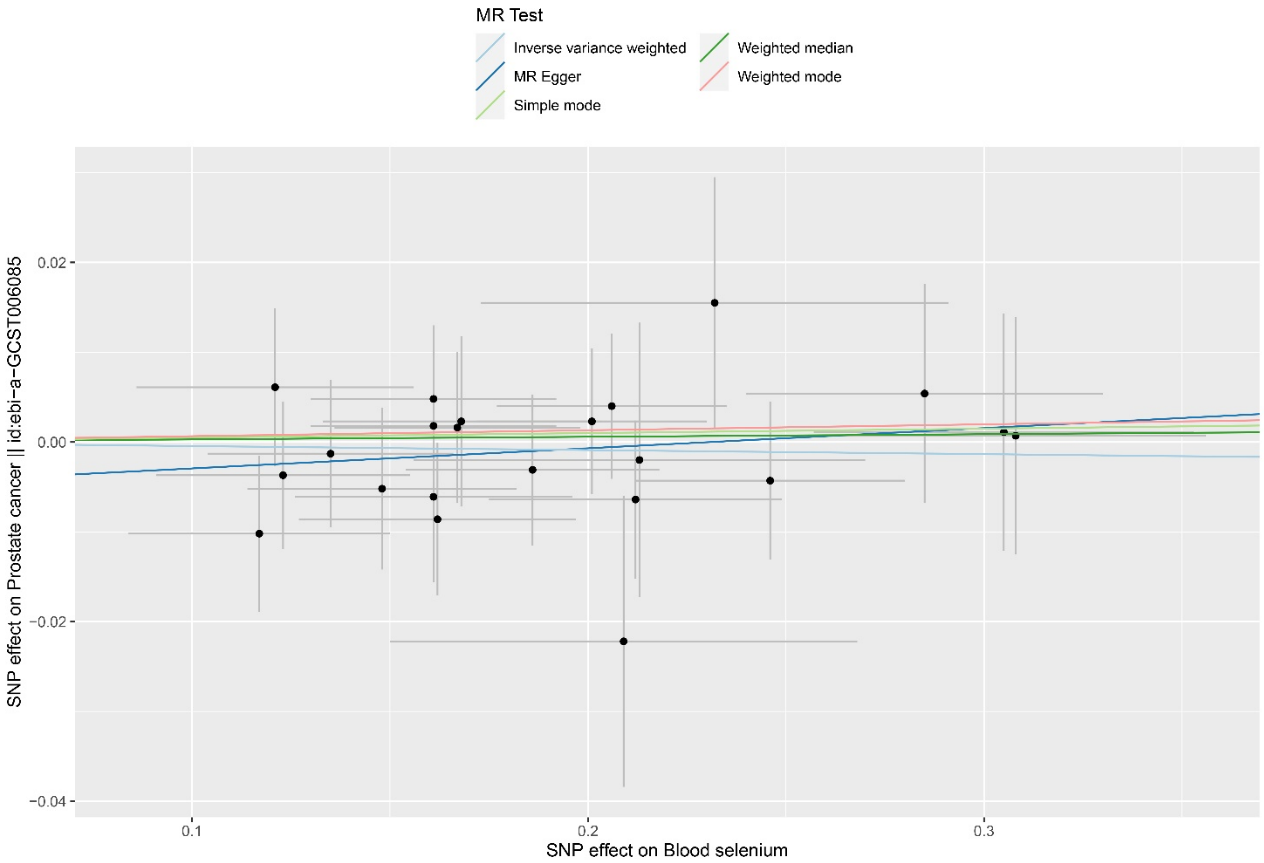

Supplementary Figure 13. Sensitivity analysis for the association between blood and toenail selenium and prostate cancer risk.

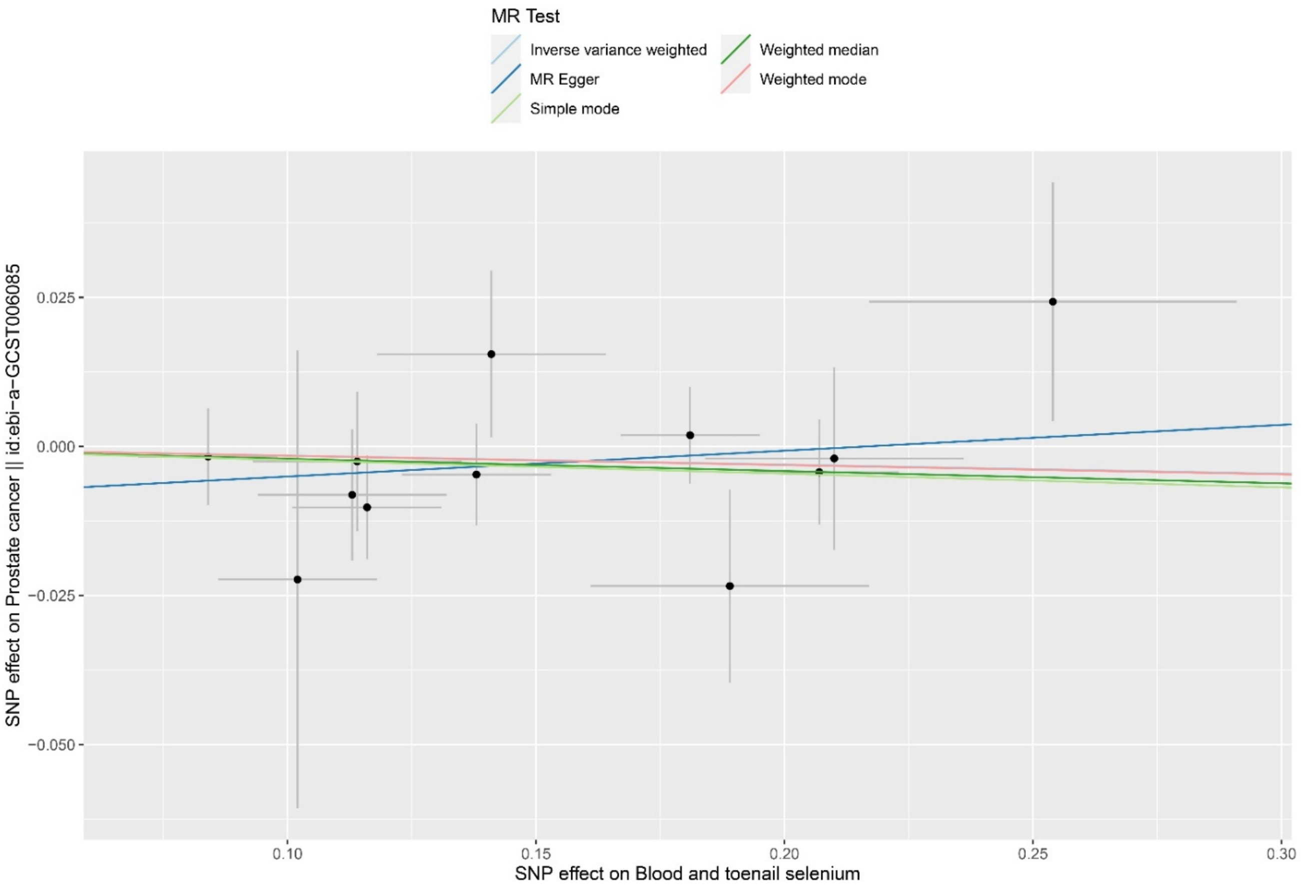

Supplementary Figure 14. Scatter plot of separate effects of SNPs for blood and toenail selenium on prostate cancer risk.

Supplementary Tables

Supplementary Table 1. SNPs selected in the study.

Items		No. of SNPs	SNPs
BCa	Iron	2	rs1525892, rs855791
	Copper	2	rs1175550, rs2769264
	Serum selenium	9	rs10514159, rs1915706, rs2445887, rs248380, rs478651, rs586199, rs9293761, rs9293769, rs949644
	Serum and toenail selenium	4	rs10944, rs234709, rs558133, rs567754
	Zinc	1	rs1532423
PCa	Iron	3	rs1525892, rs1800562, rs855791
	Copper	2	rs1175550, rs2769264
	Serum selenium	22	rs10514151, rs10514159, rs163124, rs163132, rs16876394, rs16876498, rs17823744, rs1915706, rs2445887, rs248380, rs3797535, rs478651, rs586199, rs672413, rs705415, rs7700970, rs7710824, rs8180502, rs921943, rs9293761, rs9293769, rs949644
	Serum and toenail selenium	12	rs10944, rs11951068, rs1789953, rs234709, rs3797535, rs558133, rs567754, rs6586282, rs672413, rs6859667, rs705415, rs921943
	Zinc	2	rs1532423, rs2120019
RCC (Male)	Iron	2	rs1525892, rs855791
	Copper	2	rs1175550, rs2769264
	Serum selenium	22	rs10514151, rs10514159, rs163124, rs163132, rs16876394, rs16876498, rs17823744, rs1915706, rs2445887, rs248380, rs3797535, rs478651, rs586199, rs672413, rs705415, rs7700970, rs7710824, rs8180502, rs921943, rs9293761, rs9293769, rs949644

	Serum and toenail selenium	11	rs10944, rs11951068, rs1789953, rs234709, rs3797535, rs558133, rs567754, rs6586282, rs672413, rs705415, rs921943
	Zinc	2	rs1532423, rs2120019
RCC (Female)	Iron	2	rs1525892, rs855791
	Copper	2	rs1175550, rs2769264
	Serum selenium	22	rs10514151, rs10514159, rs163124, rs163132, rs16876394, rs16876498, rs17823744, rs1915706, rs2445887, rs248380, rs3797535, rs478651, rs586199, rs672413, rs705415, rs7700970, rs7710824, rs8180502, rs921943, rs9293761, rs9293769, rs949644
	Serum and toenail selenium	12	rs10944, rs11951068, rs1789953, rs234709, rs3797535, rs558133, rs567754, rs6586282, rs672413, rs6859667, rs705415, rs921943
	Zinc	2	rs1532423, rs2120019
